# Learning to implement Smart Healthy Age-Friendly Environments

**DOI:** 10.37825/2239-9747.1021

**Published:** 2020-10-01

**Authors:** WH Van Staalduinen, JG Ganzarain, C Dantas, F Rodriguez, K Stiehr, J Schulze, C Fernandez-Rivera, P Kelly, J McGrory, C Pritchard, D Berry, M Zallio, A Ciesla, M Ulanicka, S Renaux, M Guzy

**Affiliations:** 1 AFEdemy; 2 Cáritas Diocesana de Coimbra; 3 ISIS GmbH; 4 TU Dublin; 5 Politechnika Warszawa; 6 Airelle

**Keywords:** SHAFE, training facilitators, implementation, learning

## Abstract

To develop trainings on the implementation of smart healthy age-friendly environments for people who aim to support, for example, their parents, their neighbours or local community, there are precautionary measures that have to be taken into account: the role of the facilitator (volunteer or self-employed), the level of skills, the needs of the end-users, training content and methodologies together with the sustainability of the learning. This article examines these aspects, based on desk research and expert interviews in the Smart Healthy Age-Friendly Environments (SHAFE) fields.

## I. INTRODUCTION

Healthy ageing, social inclusion and active participation can be achieved by better aligning Information and Communication Technologies (ICT) with the built and social environment: that is to realize so-called Smart Healthy Age-Friendly Environments (SHAFE). This alignment must focus on an enhancement of the user-centred design of the major concept areas associated with People (e.g. citizenship, life-long learning, social interaction in relation to their functional ability) and Places (such as houses, built environments, community spaces and outdoor facilities).

From its origin, SHAFE has its roots in the holistic age-friendly environments concept, developed by the World Health Organization in 2007[1], and further developed now into the new era of digitalization and health. This joint approach can be instrumental in arresting or slowing the progression of non-communicable diseases, promoting independent living, and thus favouring health and wellbeing.

The SHAFE-concept is currently being supported by over 200 European organisations and networks. One of the key challenges, however, is the implementation and coordination to realize SHAFE in communities and single households [2]. In our opinion, one of the main ways to foster the implementation of SHAFE is to empower citizens at local level and to provide them with informal learning methods on SHAFE measures, products, services and opportunities. The profile of these citizens is described later. The article will further focus on the needs of end-users, training content, training methods and tools to sustain the learning.

## II. METHODOLOGY

One of the focal points of this work is the *SHAFE facilitator*, a citizen who supports the active and healthy lifestyle of other people (e.g. older adults, people with impairments or disabilities), through the provision of support in different areas of life, or services and other regular wellness, lifestyle, or learning supports. They are for example volunteers, caregivers or people who see a business opportunity to deliver SHAFE products and services. Partners of the Erasmus+ project Hands-on SHAFE [3] started to investigate existing best practices to train facilitators of SHAFE, including those with lower skills or lower qualifications.

We divided the SHAFE learning modules into: SMART, HEALTHY, BUILT and BUSINESS. SMART is to learn the basics on ICT applications and which ICT is applicable to support healthy and built environments. HEALTHY focuses on lifestyle and prevention, medicines, therapies, diseases and impairments. BUILT addresses housing (new and retrofit), outdoor spaces and buildings and mobility. BUSINESS teaches how to set-up a business. Each partner investigated the current state of SHAFE in their own countries by performing desk research and interviewing both experts, using a list of structured open questions on the SHAFE fields, as well as representatives of facilitators. SHAFE reports were produced for six countries: France, Germany, Ireland, Poland, Portugal and The Netherlands. [4]. Recently it became known that Italy will use the same methodology to produce a report, facilitated by ProMIS (Programma Mattone Internazionale Salute).

## III RESULTS

Before considering detailed results in relation to training content and methods, a couple of high level observations should be made. First of all, a range of stakeholder organisations are in a position to collaborate in reaching and enabling a holistic group of potential facilitators. These include public institutions as well as nonprofit organizations or networking organizations but also, for instance, self-help groups for informal carers or neighbourhood initiatives at local level. Interest in SHAFE-oriented activities can be created by showing the possibility of an intersection between public interest and income-generating business within the Silver Economy. In addition, companies in the personal services industry or employees from providers of long-term care can be targeted. An information campaign, providing motivation or recommendations by key actors with an influential role in society are other very helpful assets to attract learners.

The main target groups of SHAFE measures (the end-users) are persons who are in need of cure or care or aiming at a healthy lifestyle to prevent potential diseases or decline due to already existing conditions. Although this covers in principle all ages and health states, older adults, persons with a disability, as well as their relatives, are the predominant targeted client groups.

Hence, in order to prepare potential and existing facilitators for their tasks, the needs of people at an advanced age or with a disability must be considered, as these volunteers, caregivers or self-employed persons will support them in (some of) their everyday needs. Problems that demand solutions at technically advanced levels will remain the domain of the subject experts. Nevertheless, retired professionals, for example, may cover this area as volunteers, too.

The role of trained SHAFE facilitators will be to personally advise the users and/or provide practical support in promoting healthy lifestyles, as well as comfortable and safe living environments. With a view to the needs of the people to be supported, facilitators must comply with the following requirements: person-centred content and methods, client oriented communication, clear and coherent information, issue targeted content, and data and privacy protection.

### A. SHAFE training content

The SHAFE training for facilitators will be developed as stand-alone modules. Each module needs to have clearly defined learning objectives, a syllabus, a method of delivery and a method of self-assessment. If the self-assessment is successfully completed, the learner is considered to have passed the course module.

SHAFE facilitators need training offerings at different levels:

- Transversal themes
- Area-specific contents
- Management and administrative know-how (in the case of starting a business)

#### a. Transversal themes

SHAFE facilitator trainees are a diverse group. As mentioned above, they can be volunteers, informal and formal caregivers, people who want to make a career switch or people who see SHAFE as a business opportunity. Besides their provision with general information on SHAFE, a personal development and training needs analysis should be performed at the very beginning. Trainees need to realize that running a business and being employed is quite different. Each participant should be enabled to recognize their motivation, strengths and training needs to compensate for missing skills. At the same time, an understanding of the context is needed.

New SHAFE facilitators who are seeking to return to paid work after e.g. time as a family carer will need a level of personal development on social awareness as their position changes. The level of self-confidence may also decrease when major changes occur with readjustments of roles.

In each particular case, facilitators need skills to assess what they can do for the client themselves and what must be left to experts in the specific area. Thus a comprehensive understanding of the characteristics, needs and fears of the clientele is indispensable. Based on that, available support from family, informal and formal caregivers and other social network members must be identified in order to act appropriately. This includes the reflection of the facilitator's own stereotypes and biases in connection to age and older people.

#### b. Area-specific contents

Potential SMART training topics were mentioned as follows:

- Awareness of the characteristics of the digital divide that cannot be expected to disappear given that the target group, e.g. older adults, may be slower in catching up with the technical developments;
- Availability of AT products and services that help ageing citizens in their daily lives, where and how to locate them, how they can improve daily life, and how they can be customised to the person’s needs, including safe and easy access;
- Identifying the level of digital literacy, confidence and support needed to operate the devices;
- Identifying and explaining available grants as well as assistance with applying for the funding;
- Informal ICT training to clients around smart phones and apps, including the selection of the most appropriate phone in terms of design, user-friendliness, the availability of a panic button, caller ID and software;
- Informal ICT training for clients around connecting smart phones to devices such as watches and pendants;
- Vulnerability awareness and assessment, e.g. around online form filling, ensuring that the form is from an official website site, never disclosing passwords.

The following contents were suggested for training for facilitators in the area of HEALTHY:

- Characteristics of ageing physiology changes with a view to the selection of services and products (e.g. sight, hearing, mobility, frailty, fine motor skills);
- Promotion of health literacy, including sleeping and eating habits;
- Opportunities to prevent diseases and possibilities for their treatment;
- Impacts of climate change on people with dementia, addiction problems or risky illnesses and possibilities of self-protection;
- Coping with persons who have been isolated, e.g. due to an illness, in order to facilitate their return back into community life;
- Assessing and promoting the personal physical fitness of older adults and people with disabilities through available online tools;
- Cognitive impairment recovery or management of progressive impairment training, e.g. by the use of games on smart phones that can be collected and selected for particular skills.

For BUILT as regards housing and public spaces a number of topics appear especially relevant:

- Available solutions in creating safe living environments according to the particular locations (living room, bathroom, bedroom, communal spaces in houses, outdoor spaces, public buildings and health facilities);
- Adapting the living environments before it is needed or as it is needed by the client; the scope of adaptations can be extensive (like bathroom renovation) or limited to very basic repairs (like changing the TV from analogue to digital transmission)
- Energy and cost savings resulting from proper insulation, insolation and installations (also using smart solutions),
- Life/design choice to avail of circular economy offers;
- Identifying and explaining grants as well as assistance with applying for funding;
- Everyday ergonomics (e.g. proper storage, tackling weights)
- Training regarding personal safety to aid the older adults to remain in their homes for longer (e.g. periodical change in daily routine to prevent or reduce opportunist crime,)
- Synchronous leisure technologies by setting up and personalizing communications technology solutions (e.g. online book clubs, cookery or fitness classes).
- Community building initiatives (e.g. social gardens, also as a part of climate change adaptation)
- Urban design supporting people with impairments (both physical and mental) to raise awareness for their needs and thus contributing to creation of co called caring communities

BUSINESS learning will focus on starting a business, developing business models and plans, financing and funding opportunities, setting up and keeping (tax) administration. The BUSINESS module will comply with existing offers in national countries for (social) entrepreneurs and business starters to take into account the big variety of national laws and measures.

#### c. Management and administrative know-how

There is an essential need for tools for facilitators in order to manage, coordinate and administer their activities. For example tools to help make and keep track of appointments, collection of notes on activities to do, collection of notes on work to be completed, collection of notes on jobs completed, boundary management, code of ethics, insurance, public funding, certification and risk analyses.

### B. Methods of SHAFE training

Training methods applied in the above-mentioned modules must be inclusive and take into account that low-qualified facilitators can be among the learners. But also for learners with a higher educational level, an easy to comprehend training design is of no disadvantage.

There are two learning paradigms to be examined. Firstly, the training to be provided to the facilitators, so they are in a position to deliver accurate and consistent support to their clients. Secondly, the training/service provided by the facilitator to the people in need of support. Successful outcomes for both learning paradigms rely on repeating the steps until the learner builds an emotional connection to the process and it becomes normal.

A successful approach in training the facilitators, and then for them in turn to train their clients, appears to be “See, Hear and Do”. According to this concept, theorized as Dale’s Cone of Experience, learners retain more information from what they “do” as opposed to what is “heard”, “read” or “observed” [5]. The contents of the training modules should be presented to the facilitators with little written text, in a visual way, using sketches and many practical examples. Also videos of not more than 3 to 4 minutes can be used with interactive material like questionnaires to handle the "do" part.

Learning steps should be small, clear and repetitive. The skill as a learning outcome must be broken down into a practice of carefully orchestrated parts of the whole skill (i.e. the part-part), to gain confidence and connection to the skill, then at the end the learner tries to perform the whole skill again.

Interactive tools such as multiple-choice tests should be used in each training module to allow for self-checks on what has been learned and what needs to be consolidated.

Strong benefits are expected for low-qualified adults from a one-to-one and face-to-face online training. One expert mentioned: "In this type of target, other types of monitoring other than personal and face-to-face during an initial learning phase are unthinkable.” An implementation of this advice in stand-alone online training modules is challenging but must be nevertheless taken seriously.

Opportunities for job shadowing, personal coaching and blended learning should be advertised. Following the education to the trainee facilitator on, for example, code of ethics and the domain in which the aid will be provided, on-the-job experiences are crucial. Opportunities in which the trainee facilitator is guided while working on the job with an experienced mentor and building up the skills of interacting and listening to clients must be collected and recommended in order to ensure a sustainable training outcome.

For volunteers, 60 learning hours of 45 minutes were considered the upper limit [6]; this is in accordance with, for instance, the duration of the courses for licenced trainers in preventive sports programmes in Germany. Professional advice and the sharing of experiences once a week should be arranged for at least six months after the training.

### C. SHAFE sustainable training

A couple of measures are important to sustain the activities of SHAFE facilitators. For some, a principle distinction should be made between volunteers and persons who wish to start their own business. Some measures, however, apply to both of the groups.

Firstly, it appears useful to cooperate with local partners in order to foster the SHAFE training packages and the implementation of measures. Organizations with an important territorial anchorage and activities impacting smart, healthy age-friendly environments, who are well-known and effective in meeting the main needs of end-users and facilitators qualify as preferred partners in this context. Municipalities, in particular, should be encouraged to provide funding or support for SHAFE facilitators in terms of advertising their services, especially those affected by outflow of the young and experiencing an accelerated process of population ageing. Such municipalities are also often hit by economic difficulties, which are illustrated by high unemployment rate. It is vital therefore to give their inhabitants tools to enable them to find new paths of economic activity.

In addition, health centres, NGOs, family doctors and other stakeholders in touch with potential clients that need SHAFE products or services, as well as grocery stores, cafes and kiosks, can take on important functions in disseminating the facilitation services.

Facilitators will feel more connected if they operate in a system. On the side of the end-users, there is a need for a trusted network, marketplace or central point of contact to find out and negotiate about services and products and for localised support of products and services. Belonging to such a network would lend credibility to a facilitator externally. Internally, it would provide the opportunity for registered facilitators to support each other and share their experiences. Also monitoring and gathering feedback from users could be facilitated by such a network.

Organizations that run measures with volunteers must make sure that activities are neither surpassing their professional capacities nor embracing too many unpleasant activities. For instance, volunteers should not be expected to do the job of a professional caregiver, for example the allocation of medicine or changing diapers. Volunteers should at least be provided with the following support: logistical provisions, organisational support and provisions and support in public relations.

## IV. CONCLUSIONS

Learning of SHAFE measures takes the needs of end-users into account. Predominant targeted client groups are older adults and people with a disability. We further learned that the training content for facilitators needs to be developed as stand-alone modules; each of them must have clearly defined learning objectives, a syllabus, method of delivery and method of self-assessment. We also recognised that facilitators may have many different backgrounds and therefore different methodologies are needed. Besides focusing on the content of learning, it is also crucial to take into account the learning of certain transversal themes. By these themes, the facilitators learn more about their own attitude and prejudices, appearance and communication skills, essential features for people who want to act as a volunteer or self-employed SHAFE service provider and crucial to support the implementation of Smart Healthy Age-Friendly Environments at local level in Europe. Environments that at the end benefit wellbeing, participation and healthy ageing of all citizens.
